# Graphical Workflow System for Modification Calling by Machine Learning of Reverse Transcription Signatures

**DOI:** 10.3389/fgene.2019.00876

**Published:** 2019-09-25

**Authors:** Lukas Schmidt, Stephan Werner, Thomas Kemmer, Stefan Niebler, Marco Kristen, Lilia Ayadi, Patrick Johe, Virginie Marchand, Tanja Schirmeister, Yuri Motorin, Andreas Hildebrandt, Bertil Schmidt, Mark Helm

**Affiliations:** ^1^Institute of Pharmacy and Biochemistry, Johannes Gutenberg-University, Mainz, Germany; ^2^Institute of Computer Science, Scientific Computing and Bioinformatics, Johannes Gutenberg-University, Mainz, Germany; ^3^Institute of Computer Science, High Performance Computing, Johannes Gutenberg-University, Mainz, Germany; ^4^Next-Generation Sequencing Core Facility UMS2008 IBSLor CNRS-UL-INSERM, Biopôle, University of Lorraine, Vandœuvre-lès-Nancy, France; ^5^IMoPA UMR7365 CNRS-UL, Biopôle, University of Lorraine, Vandœuvre-lès-Nancy, France

**Keywords:** RT signature, Watson–Crick face, Galaxy platform, RNA modifications, machine learning, m1A

## Abstract

Modification mapping from cDNA data has become a tremendously important approach in epitranscriptomics. So-called reverse transcription signatures in cDNA contain information on the position and nature of their causative RNA modifications. Data mining of, e.g. Illumina-based high-throughput sequencing data, is therefore fast growing in importance, and the field is still lacking effective tools. Here we present a versatile user-friendly graphical workflow system for modification calling based on machine learning. The workflow commences with a principal module for trimming, mapping, and postprocessing. The latter includes a quantification of mismatch and arrest rates with single-nucleotide resolution across the mapped transcriptome. Further downstream modules include tools for visualization, machine learning, and modification calling. From the machine-learning module, quality assessment parameters are provided to gauge the suitability of the initial dataset for effective machine learning and modification calling. This output is useful to improve the experimental parameters for library preparation and sequencing. In summary, the automation of the bioinformatics workflow allows a faster turnaround of the optimization cycles in modification calling.

## Introduction

In the rapidly growing field of epitranscriptomics (Saletore et al., 2012), the detection of RNA modifications is typically based on a combination of reagents and tools for wet work on the one hand, and bioinformatics processing of massive amounts of RNA-Seq data, on the other hand. Because of a sequence space that may include up to 10^7^ nucleotides and more, transcriptomes must be scrutinized by computer-assisted detection schemes, resulting in what is called modification calling (Helm and Motorin, 2017).

With the exception of the up-and-coming nanopore direct RNA sequencing technology (Byrne et al., 2017; Garalde et al., 2018; Smith et al., 2019), RNA-Seq data are obtained after reverse transcription of the modified RNA template into DNA, a process during which information about modification type and position may get erased, partially or completely, since the newly synthesized cDNA is composed only of the four canonical deoxynucleotides. Attempts to circumvent this problem included, for example, the use of various chemical reagents, which specifically react with a given modification, to alter cDNA synthesis at sites of RNA modifications. One such reagent is CMCT, a carbodiimide leading to stalling of cDNA synthesis at sites of pseudouridine modification in the RNA template (Ofengand and Bakin, 1997; Carlile et al., 2014; Schwartz et al., 2014). Other modifications do not require chemical derivatization to alter cDNA synthesis. In particular, modifications with chemical alterations on their Watson–Crick face are liable to cause cDNA synthesis differing from that expected of an unmodified RNA template. A case in point is m^1^A, a modification featuring a methyl group on the Watson–Crick face of adenosine, which interferes with proper base pairing, in RNA structure (Helm et al., 1998) (Helm et al., 1999) (Lempereur et al., 1985; Zhou et al., 2016), as well as during cDNA synthesis by reverse transcription (Motorin et al., 2007). In the particular case of m^1^A, the resulting cDNA was shown to contain products of transcription arrest, i.e. abortive cDNA fragments, as well as misincorporation, most frequently of dATP being incorporated instead of dTTP at the position corresponding to the modification site. The ensemble of erroneous events in cDNA synthesis has been termed *reverse transcription signature* and was shown to depend on a number of factors including e.g. the nature of the penultimate base encountered by the RT enzyme before engaging the modified RNA residue (Hauenschild et al., 2015). The RT signature of m^1^A can be experimentally altered e.g. by enzymatic demethylation with the AlkB enzyme (Zheng et al., 2015; Liu et al., 2016; Li et al., 2017) or at alkaline pH, which induces a Dimroth rearrangement to m^6^A (Dominissini et al., 2016; Safra et al., 2017). Since these processes are relatively specific to m^1^A, they can be exploited to increase confidence in modification calling, therein being used as a validation (Helm and Motorin, 2017).

All of the above processes require significant computing efforts to extract information on RNA modifications from RNA-Seq data. Given that the composition of RT signature of a given modification in terms of RT arrest, misincorporation, and even template nucleotide skipping (“jumps”) (Ebhardt et al., 2009; Findeiss et al., 2011; Ryvkin et al., 2013; Hauenschild et al., 2015) is subject to variations caused by factors that are not fully characterized and thus cannot be entirely controlled, an innovative approach to account for a maximum of these features and exploit them for computer-based prediction (“modification calling”) involves machine learning. A particular brand of machine learning, the random forest, was used for the purpose of modification by several groups, including us (Hauenschild et al., 2015).

**Figure 1:**
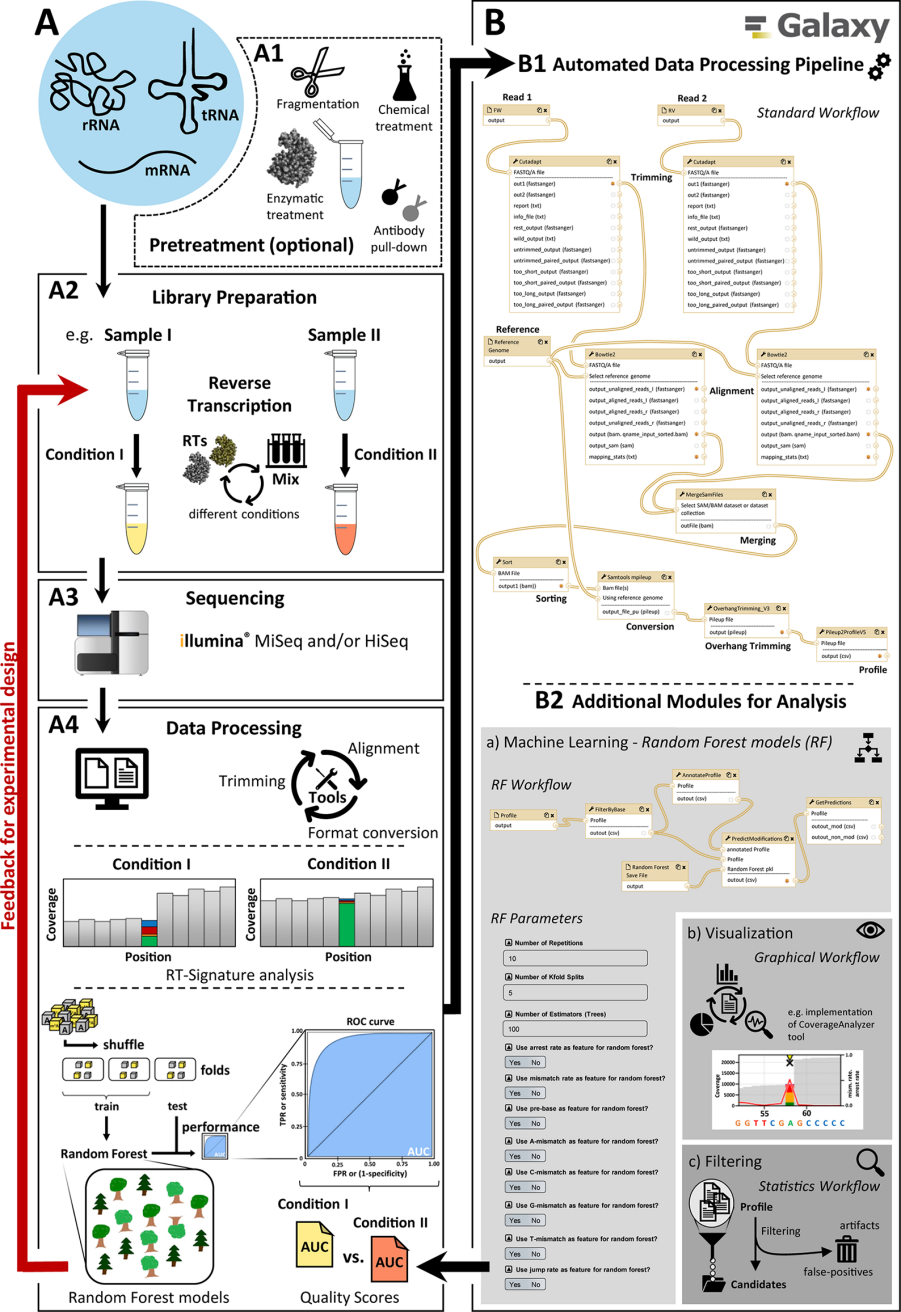
Main overview of the modification calling pipeline. A diagram showing the different steps for creating and analyzing RNA-Seq data. The pipeline has two parts: **(A)** general workflow for the processing of RNA samples and **(B)** the implemented automated graphical workflow system with the available modules for bioinformatics data analysis. **(A)** consists of (A1) possible and partly necessary pretreatments for different RNA species, (A2) library preparation with the possibility of adaptations (e.g. conditions for reverse transcription), (A3) sequencing with Illumina sequencing platforms (e.g. MiSeq/NextSeq and HiSeq), and (A4) data processing including basic data treatment like adapter trimming, alignment, and format conversion, as well as data analysis (e.g. machine learning and RT-signature analysis). The elaborate data processing (A4) was fully automated in **(B)** by using the open-source Galaxy platform to create and provide a quick and user-friendly feedback mechanism to optimize the experimental design, sample preparation, and data processing. The standard workflow (B1) is supplemented by various additional modules (B2) including workflows for (a) machine learning, (b) visualization, and (c) filtering.

Optimizing the performance of a modification calling protocol requires multiple rounds, beginning with a wet work part of library preparation and subsequent Illumina sequencing, as illustrated in **Figure 1A**. Here, a pretreatment (A1) of the samples by using auxiliary reagents such as the demethylase AlkB or changes in the library preparation part (A2), e.g. by employing different reverse transcriptase enzymes or variegated reaction conditions, are implemented experimentally. After sequencing (A3), a fast evaluation of their influence on the RT signature and consequently on RF performance (A4) is necessary to proceed with the next round of optimized library preparation in the wet lab. The associated computational data mining thus represents a bottleneck on the path to optimal modification calling.

To address this shortcoming, we here present an automated workflow implementation based on Galaxy (Afgan et al., 2018), whose components are depicted in **Figure 1B**. The Galaxy implementation provides a first module (B1) for the automation of typical and recurrent RNA-Seq–associated operations such as trimming and mapping. While these operations can be customized to accommodate a range of data formats, it allows procedurally stable and reproducible treatment of data package of comparable content, such as RNA-Seq data obtained under variegated conditions for library preparation. This, in turn, allows a comparative evaluation of those experimental conditions, as outlined above. The same holds true for subsequent modules (B2), designed and implemented following the requirement for fast comparison of data packages. The implemented tools allow to quantify mismatch, jump, and arrest rates in the relevant transcriptome, thus compiling RT signatures at single-nucleotide resolution. Still automatized, RT signatures of modified RNA nucleotides can be transferred as positive instances for machine learning, along with negative instances, i.e. signatures of unmodified nucleotides. Positive and negative instances are then used to train a Python-based random forest implementation of machine learning, and the performance of the trained machine in modification calling is evaluated and reported as a feedback in a further round of experimental optimization. Finally, with the implementation of a visualization module, graphics can be displayed and extracted for visual examination and comparison of individual sequence segments as well as the entire RNA fragments in a publishable manner.

## Materials and Methods

### RNA Sequencing Analysis

The present workflow serves as the main process for the analysis of RNA sequencing data in respect to the detection of several modifications. Its Galaxy distribution comes with a number of adjustable elements for variegated workflows, in which the particular element (Workflow *RNA_Seq_Standard_Workflow*) serves as basis for the remaining workflows and functionalities. Therefore, it is referred to as “standard workflow.” The overall scheme of the workflow is illustrated in **Figure 1** (B1) and consists of the following steps:

#### Preprocessing of Raw Reads (Trimming)

The raw reads from the sequencing data (stored in fastq-format) are first subjected to removal of auxiliary sequences such as adapters, barcodes, and unique molecular identifiers (UMIs). For this task, the workflow uses the Cutadapt trimming software (Martin, 2011). Due to the necessity to remove multiple sequences from the raw reads, their respective arrangement, and the configuration of Cutadapt, the trimming is separated into multiple steps. In a typical Illumina paired-end sequencing run, the forward and reverse reads are stored in individual fastq files; the reads show slightly different characteristics concerning the auxiliary elements; hence, the trimming for forward and reverse reads is performed separately. The first substep in the trimming process consists of the removal of Illumina adapter sequences. In a second step, terminal barcode sequences and UMIs (Miner et al., 2004; McCloskey et al., 2007; Casbon et al., 2011) are cut from the raw reads.

#### Alignment

Mapping to a given sequence reference file is performed with Bowtie 2 (Langmead and Salzberg, 2012). Again, this process is performed separately for forward and reverse reads (–nofw/–norc option) and therefore in single-end mode. For the detection of RT-impairing modifications like m^1^A, it is necessary to allow for mismatches (One mismatch [“N1”] allowed in seed length of 6 [“L6”]). Values are tailored toward tRNAs (e.g. high amounts of RT-impairing modifications). Additionally, if the evaluation is performed on samples containing a large number of modifications (affecting the RT), the amount of allowed mismatch occurrences has to be increased by adjusting the seed-length option (Bowtie standard parameters allow for one mismatch within a given seed; hence, seed length has to be decreased for highly modified samples). The alignment is stored in BAM format.

The two BAM files, one for the forward and one for the reverse reads correspondingly, are merged using the SAMtools (Li et al., 2009) “merge” function, and the aligned reads are sorted according to chromosomal coordinates.

#### File Conversion and Overhang Trimming

Further analysis steps require information of mapped reads at single base resolution for each position in the reference sequence, as every position is evaluated for mismatch and arrest properties. Accordingly, the BAM-file is converted into Pileup-format using the SAMtools (Li et al., 2009) “mpileup” function. As described in Tserovski et al. (2016), the library preparation includes a step in which C-tailing at the 3′ end of the cDNA strand was performed. Due to this tailing step in the library preparation protocol, despite the previous trimming steps, some tailing bases (overhangs) can remain and were then aligned with the reads. As these overhangs can impede the detection of modified sites, they have to be removed from the alignment. Therefore, a Python-based algorithm for postalignment manipulation was developed. This algorithm finds read-ending bases and compares them to reference base and removes them in case of a mismatch. After the overhang trimming, the data are still stored in Pileup format.

#### Feature Extraction

Information on each position of the reference is then extracted from the Pileup format and subsequently stored in a format termed “Profile” (example shown in **Table 1**). The information consists of the following features:

**Arrest rate:** Drop in coverage in relation to the preceding (N+1) position (arrest).
**Mismatch rate:** Relative amount of mapped nucleobases not matching the respective base in the reference (mismatch).
**Jump rate:** Relative amount of deletions (bases left out during reverse transcription) occurring at the given position in the reference (jump). A distinction is made between deletions at the given position in the reference (single jumps direct), deletions at the neighboring position (−1 position) (single jump delayed), and deletions at the given position, as well as the neighboring position (double jump).

**Table 1:** Extracted Profile file after filtering with *Demethylation_relative_change* module with all m^1^A candidate positions.

ref_seg	pos	refbase	cov	prebase	mismatch	A	G	T	C	N	a	g	t	c	n	single_jump_direct	single_jump_delayed	double_jump	arrest
tdbR00000370|Saccharomyces_cerevisiae|4932|Arg|TCT	57	A	699	C	0.29471	493	8	2	94	0	0	5	5	92	0	0.00000	0.02710	0.00285	0.10941
tdbR00000300|Saccharomyces_cerevisiae|4932|Asn|GTT	59	A	961	C	0.37045	605	7	6	125	0	0	7	69	142	0	0.00000	0.00407	0.02238	0.15544
tdbR00000021|Saccharomyces_cerevisiae|4932|Cys|GCA	57	A	405	T	0.21728	317	13	39	0	0	0	7	28	1	0	0.00000	0.00000	0.00000	0.43399
tdbM00000003|Saccharomyces_cerevisiae|4932|Gln|TTG	57	A	475	A	0.15789	400	11	18	1	0	0	12	29	4	0	0.00000	0.00000	0.00000	0.26810
tdbR00000170|Saccharomyces_cerevisiae|4932|Ile|AAT	59	A	919	T	0.38085	569	55	88	6	0	0	67	127	7	0	0.00429	0.00000	0.01072	0.15350
tdbM00000006|Saccharomyces_cerevisiae|4932|Ile|TAT	58	A	373	T	0.25469	278	13	28	4	0	0	7	34	9	0	0.00000	0.00000	0.00000	0.31934
tdbR00000192|Saccharomyces_cerevisiae|4932|Lys|CTT	58	A	2715	G	0.16317	2272	102	103	9	0	0	108	112	9	0	0.00037	0.00000	0.00293	0.07658
tdbR00000193|Saccharomyces_cerevisiae|4932|Lys|TTT	58	A	619	G	0.43942	347	49	75	10	0	0	62	68	8	0	0.00478	0.00000	0.00955	0.16511
tdbR00000323|Saccharomyces_cerevisiae|4932|Pro|TGG	57	A	459	T	0.43573	259	3	69	0	0	0	12	112	4	0	0.00000	0.00000	0.00000	0.18905
tdbR00000324|Saccharomyces_cerevisiae|4932|Pro|TGG	57	A	439	T	0.43508	248	4	56	1	0	0	9	121	0	0	0.00000	0.00000	0.00000	0.20364
tdbR00000443|Saccharomyces_cerevisiae|4932|Thr|AGT	58	A	396	A	0.28283	284	23	23	3	0	0	28	30	5	0	0.00000	0.00222	0.12195	0.38608
tdbR00000444|Saccharomyces_cerevisiae|4932|Thr|AGT	58	A	616	A	0.31656	421	39	47	5	0	0	41	54	9	0	0.00145	0.00000	0.10320	0.30152
tdbR00000464|Saccharomyces_cerevisiae|4932|Val|AAC	59	A	1066	T	0.18386	870	33	55	22	0	0	18	61	7	0	0.00187	0.00000	0.00094	0.69026

In addition, the reference name (ref seg), reference base (refbase), reference position (pos), and coverage at the respective position (cov) are stored in the Profile. Also included is detailed information on the alignment numbers for each type of base (A, C, G, T) and unknown read bases (N), as well as the type of base preceding the position (prebase) in question.

In many cases, modified positions heavily differ from nonmodified positions in these key characteristics. Nonmodified bases are not expected to cause arrest and mismatch signals (at least not at high levels), making these features a main target for differentiation between modified and unmodified sites.

### Downstream Analysis

The generation of the Profile file concludes the standard workflow. From this point on, the proceedings heavily vary depending on the question being investigated, with the Profile file serving as the starting point. Options for downstream analysis are shown in **Figure 1** (B2) and include the following:

#### Filtering

An option for further evaluation is a simple filtering process. Here, adenosine instances can be separated into two categories, namely, “likely m^1^A” and “likely non-m^1^A.” The selectable filter criteria include threshold values for mismatch and arrest rates, minimum coverage, and the nucleobase of interest. In most cases, the arrest and mismatch rates should be sufficient to separate m^1^As from non-m^1^As.

**Figure 2:**
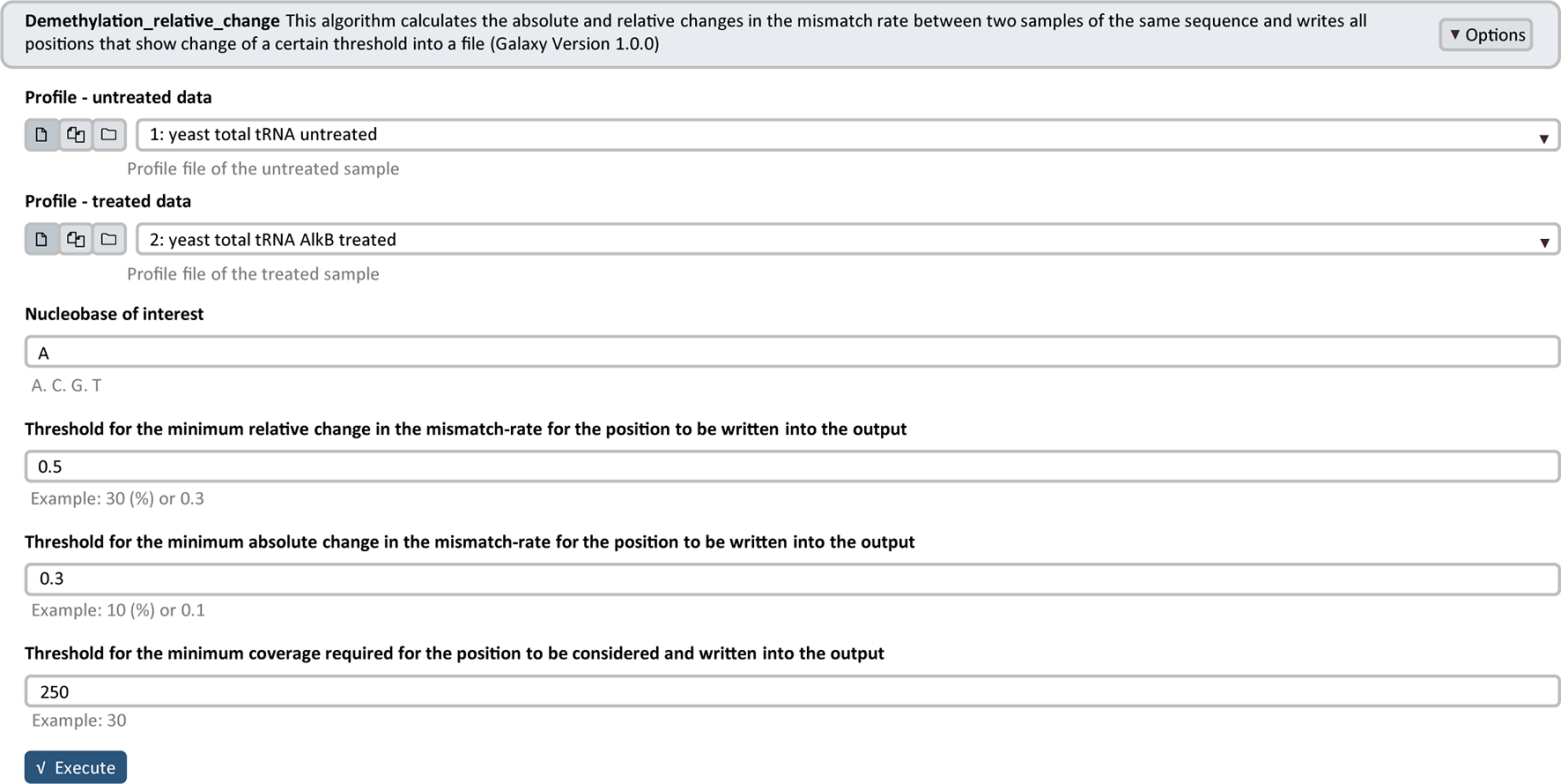
Galaxy Filtering module *Demethylation_relative_change* interface. As input, two Profile files, yeast total tRNA untreated and yeast total tRNA AlkB treated, are used with the following selected parameters for filtering: adenosine (A) as nucleobase of interest, 0.5 or 50 (%) and 0.3 or 30 (%) as thresholds for the minimum relative and absolute changes in the mismatch rate and 250 as threshold for the minimum coverage required.

Another filtering option includes the comparison of two samples after different treatment. In our Galaxy pipeline, the sample comparison after enzymatic or chemical treatment is implemented wherein one sample serves as a reference (**Figure 2**). The algorithm calculates the absolute and relative changes in the mismatch rate between 2 samples for each position and filters by means of adjustable thresholds for changes and coverage. The resulting Profile file contains candidates filtered according to the selected thresholds. This module can be used for verification of modification candidates by e.g. applying enzymatic or chemical treatment to remove the alterations at the Watson–Crick face impeding reverse transcription and therefore decreasing the mismatch rate (exemplary analysis shown in Results section).

#### Machine Learning

For the prediction of m^1^A and other modifications, a machine learning model for binary classification is included in the Galaxy distribution (Workflow *Workflow_Prediction*). The associated workflows for training and prediction are based on a random forest model from the “scikit-learn” Python package (Pedregosa et al., 2011). For the training process, the positive class (modified bases) and negative class (nonmodified bases) are given as input in a 1:1 ratio. This ratio is used in order to counter the tendency of RF models to bias toward the majority class. This RF property frequently leads to false negatives for the positive class (the modifications) when making predictions. Importantly, this bias is not necessarily reflected by the evaluation scores. The random forest performs e.g. 10 repetitions of a 5-fold cross-validation. These parameters can be adjusted as required for different models. The model’s performance is measured by the area under the receiver operating characteristic curve. A detailed description of the concept of the random forest model used for this workflow can be found in Hauenschild et al. (2015). The prediction workflow requires a trained random forest model and a Profile file as input and performs a binary classification.

#### Visualization

**Figure 3:**
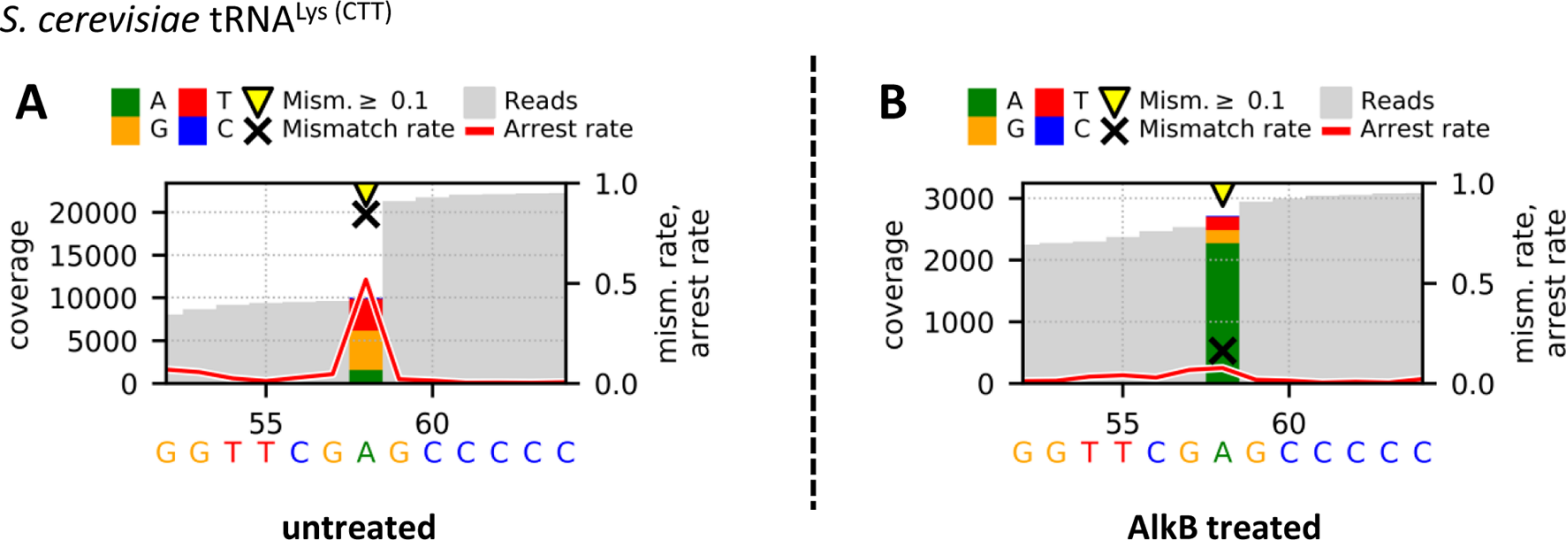
Graphical plots of untreated **(A)** and AlkB-treated **(B)** yeast tRNA^Lys (CTT)^ using the additional module *Visualize_V3* for visualization. Sites with error rates of more than 10% are highlighted with yellow arrows, with colored bars indicating the nature of the reads. Mismatch rates are depicted as black crosses, and arrest rates as red lines. The m^1^A site is located in the middle of the shown sequence segment at position 58.

A graphical representation of the position of interest within sequence context can be created using a Python-based script (Workflow *Visualize_V3*), extracted from the CoverageAnalyzer tool (Hauenschild et al., 2016). The user can plot a sequence containing up to 1000 bases where the leftmost and rightmost bases can be selected by position. In addition, various sizes can be adjusted, including the width and height of the plot, the font size, and the size of markers within the graphic (exemplary plot shown in **Figure 3**).

### RNA Sequencing—Sample Preparation

#### Library Preparation and Sequencing

Sample preparation and sequencing are performed according to a previously published protocol (Hauenschild et al., 2015; Tserovski et al., 2016). This library preparation protocol includes the possibility to catch abortive products during the reverse transcription step, important for the detection of modifications impeding reverse transcription and generating a certain amount of RT stop products. The protocol also allows the adaptation of almost all necessary steps for preparation of RNA-Seq libraries, including adapter ligations, reverse transcription, and polymerase chain reaction. This allows fast screening of different conditions during sample preparation. Special experimental changes (e.g. buffer changes or pretreatment of the RNA) during library preparation for the preparation of our shown exemplary data are mentioned in the Results section.

## Results

### Enzymatic Demethylation of m^1^a Sites in Yeast tRNA With AlkB

In an exemplary sample processing, two samples of total tRNA from *Saccharomyces cerevisiae* were used for sample preparation, sequencing, data processing, and analysis. One of the samples had been subjected to pretreatment (**Figure 1** [A1]) with α-ketoglutarate–dependent dioxygenase AlkB that “repairs” alkylated DNA and RNA containing 3-methylcytosine (m^3^C) or 1-methyladenine (m^1^A) by oxidative demethylation. Protein preparation and sample treatment were performed according to a previously published protocol (Zheng et al., 2015). The second sample was used as reference. Both samples were then used as starting material for library preparation and subsequent sequencing (**Figure 1** [A2, A3]). Library preparation and sequencing were performed as described in our published workflow by Hauenschild et al. (2015) and Tserovski et al. (2016). The sequencing output data packages in FASTQ format were then processed with the standard automated Galaxy workflow *RNA_Seq_Standard_Workflow* (**Figure 1** [B1]) to create Profile files for downstream analysis.

### Filtering for Demethylation Candidates

The Profile files were used for statistical analysis. **Figure 2** illustrates the Galaxy Filtering module *Demethylation_relative_change*, which was used to filter and extract all positions that show an absolute and relative change in the mismatch rate of a certain threshold between the untreated and AlkB-treated sample. **Table 1** shows the extracted Profile file with all candidate positions after filtering. From our sample comparison, with our selected thresholds, 13 candidate positions fulfilling the requirements were filtered out, with high probability to be m^1^A sites.

### Visualization of Demethylation Candidates

In addition, the Profile files were used in the visualization workflow *Visualize_V3* to obtain graphical plots for each sample. The visual comparison of the untreated (A) and AlkB-treated (B) yeast tRNA^Lys (CTT)^, which includes an m^1^A at position 58, is shown in **Figure 3**. The strong decreases of the mismatch and arrest rate from 0.845 and 0.518 to 0.163 and 0.077 after AlkB treatment at position 58 of the shown sequence segment indicate a successful removal of the methylation and therefore enabled valid reverse transcription. Such changes in the reverse transcription signature are considered as effective validation of the actual presence of m^1^A at the considered position.

### Influence of Mn^2+^ on the RT Signature at m^1^A Sites in Yeast tRNA

In a second exemplary sample processing, four samples of total tRNA from *S. cerevisiae* were used for sample preparation, sequencing, data processing, and analysis. The samples were used for library preparation and differed in the reverse transcription step (**Figure 1** [A2]). For reverse transcription, we used SuperScript^®^ III Reverse Transcriptase (Thermo Fisher Scientific, Germany) in four different buffer mixtures to investigate the influence of Mn^2+^ during reverse transcription (Zhou et al., 2018). Sample A served as a reference and was prepared according to the supplier’s manual, using the standard RT buffer with Mg^2+^. For the other three test samples, custom-made RT buffers, including the standard buffer components, and Mn^2+^ in different concentrations (0.5 mM [B], 1.0 mM [C] or 3.0 mM [D]) instead of Mg^2+^, were used. Library preparation and sequencing were performed as described in our published workflow by Hauenschild et al. (2015) and Tserovski et al. (2016). The sequencing output data packages in FASTQ format were then processed with the standard automated Galaxy workflow *RNA_Seq_Standard_Workflow* (**Figure 1** [B1]) to create Profile files for downstream analysis.

### Visualization of tRNA^Asn (GTT)^ Using Mg^2+^ or Mn^2+^ as Buffer Components for Reverse Transcription During Library Preparation

**Figure 4:**
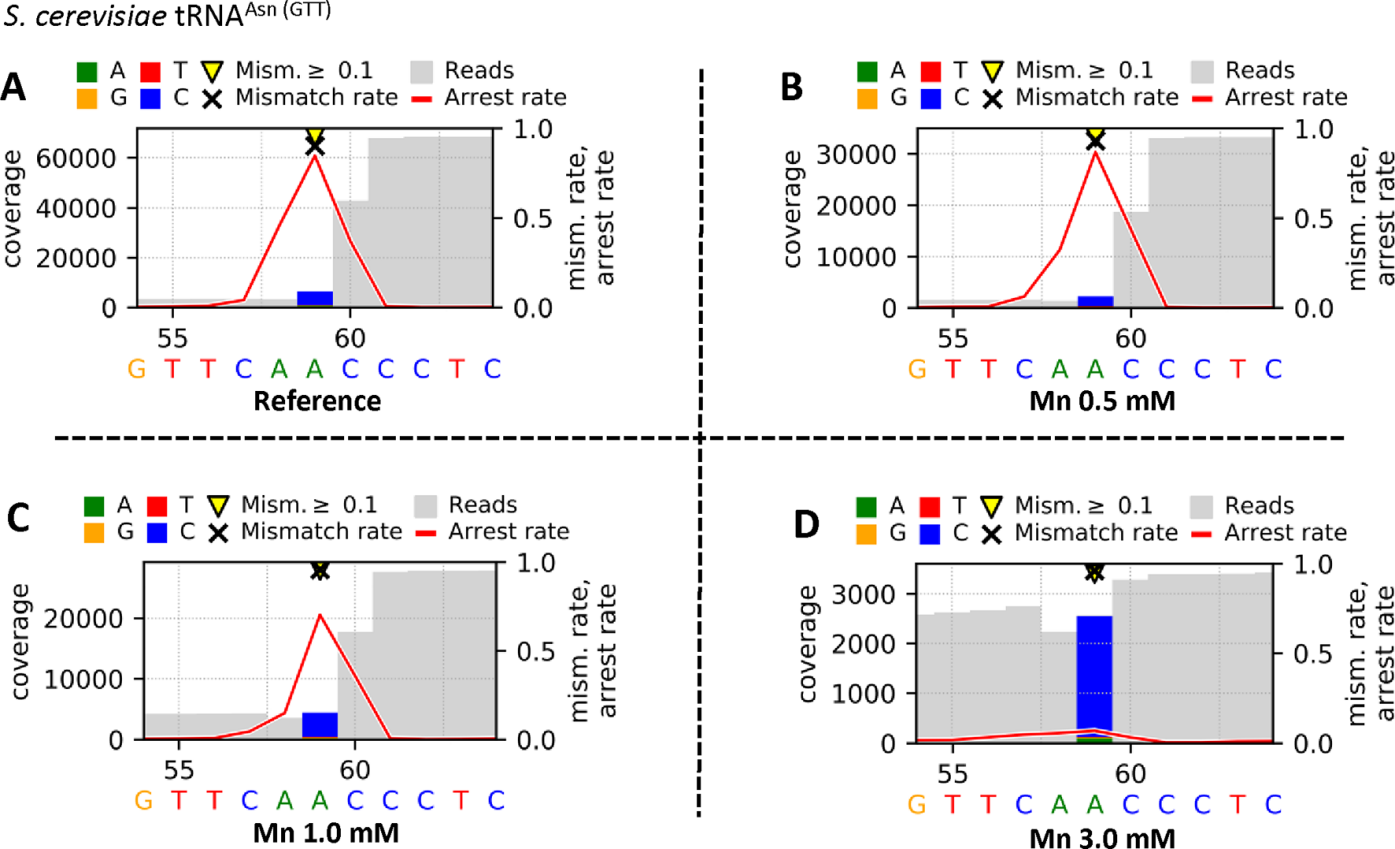
Graphical plots of yeast tRNA^Asn (GTT)^, which was used for library preparation, visualized by using the additional module *Visualize_V3*. The reverse transcription step was performed by using SuperScript^®^ III Reverse Transcriptase in different reaction buffers. The supplier’s standard reaction buffer (First Strand Synthesis buffer) with Mg^2+^ serves as reference **(A)**, and the tested buffer mixtures differ by increased concentrations of Mn^2+^ [0.5 mM **(B)**, 1.0 mM **(C)**, 3.0 mM **(D)**] as Mg^2+^ substitute. Sites with error rates of more than 10% are highlighted with yellow arrows, with colored bars indicating the nature of the reads. Mismatch rates are depicted as black crosses, and arrest rates as red lines. The m1A site is located in the middle of the shown sequence segment at position 59.

The Profile files were used in the visualization workflow *Visualize_V3* to obtain graphical plots for each sample. The visual comparison of the reference (**Figure 4A**) and the Mn^2+^ (0.5 mM [**Figure 4B**], 1.0 mM [**Figure 4C**], or 3.0 mM [**Figure 4D**]) yeast tRNA^Asn (GTT)^ samples, including an m^1^A at position 59, is shown in **Figure 4**. The high mismatch rates (≥90%) throughout all samples are driven by the prebase influence (Hauenschild et al., 2015), leading to a consistently high C mismatch. Considering the m^1^A at position 59, the strong decrease in the arrest rate at position 59 from 0.846 (A) over 0.869 (B) and 0.704 (C) down to 0.070 (D) indicates an increasing read-through capability of the reverse transcriptase due to a stabilizing effect by increased Mn^2+^ concentrations. In addition, by exchanging Mg^2+^ through Mn^2+^, the number of jumps (single_jump_direct, single_jump_delayed, double_jump) increases with higher Mn^2+^ concentrations, visible in **Table 2**, as well as in the graphical plots by coverage drops (through deletions/jumps), especially visible in **Figure 4D**.

**Table 2:** Extracted Profile data for yeast tRNA^Asn (GTT)^ after library preparation with 4 different buffer mixtures for the reverse transcription step. Shown are data for positions 58, 59 (m^1^A), and 60.

ref_seg	pos	refbase	cov	prebase	mismatch	A	G	T	C	N	a	g	t	c	n	single_jump_direct	single_jump_delayed	double_jump	arrest
tdbR00000300|Saccharomyces_cerevisiae|4932|Asn|GTT Reference	58	A	3238	A	0.02471	3158	4	4	33	2	0	5	7	25	0	0.01927	0.00056	0.00000	0.4574
tdbR00000300|Saccharomyces_cerevisiae|4932|Asn|GTT 0.5 mM Mn	58	A	1380	A	0.04855	1313	4	1	47	3	0	2	0	10	0	0.02404	0.00000	0.00060	0.32355
tdbR00000300|Saccharomyces_cerevisiae|4932|Asn|GTT 1.0 mM Mn	58	A	3546	A	0.04061	3402	15	9	79	0	0	13	6	22	0	0.02913	0.00000	0.00067	0.14965
tdbR00000300|Saccharomyces_cerevisiae|4932|Asn|GTT 3.0 mM Mn	58	A	2239	A	0.04332	2142	9	6	37	7	0	12	5	21	0	0.05623	0.00172	0.00138	0.0565
tdbR00000300|Saccharomyces_cerevisiae|4932|Asn|GTT Reference	59	A (m^1^A)	6311	C	0.90160	621	79	36	3431	6	0	119	25	1994	0	0.00000	0.01048	0.04161	0.84647
tdbR00000300|Saccharomyces_cerevisiae|4932|Asn|GTT 0.5 mM Mn	59	A (m^1^A)	2210	C	0.93167	151	37	59	1238	8	0	37	15	665	0	0.00041	0.01630	0.09902	0.86879
tdbR00000300|Saccharomyces_cerevisiae|4932|Asn|GTT 1.0 mM Mn	59	A (m^1^A)	4454	C	0.95757	189	65	95	2208	1	0	75	35	1786	0	0.00038	0.02481	0.14907	0.70422
tdbR00000300|Saccharomyces_cerevisiae|4932|Asn|GTT 3.0 mM Mn	59	A (m^1^A)	2568	C	0.96145	99	9	9	1149	14	0	7	5	1276	0	0.00000	0.05323	0.16101	0.06965
tdbR00000300|Saccharomyces_cerevisiae|4932|Asn|GTT Reference	60	C	42890	C	0.00445	87	30	22	42699	21	20	10	1	0	0	0.00000	0.00000	0.00000	0.36943
tdbR00000300|Saccharomyces_cerevisiae|4932|Asn|GTT 0.5 mM Mn	60	C	18703	C	0.00733	51	12	10	18566	50	11	3	0	0	0	0.00000	0.00005	0.00000	0.43528
tdbR00000300|Saccharomyces_cerevisiae|4932|Asn|GTT 1.0 mM Mn	60	C	17706	C	0.00345	17	7	10	17645	10	9	6	2	0	0	0.00006	0.00011	0.00011	0.35852
tdbR00000300|Saccharomyces_cerevisiae|4932|Asn|GTT 3.0 mM Mn	60	C	3287	C	0.01156	2	1	9	3249	14	5	5	2	0	0	0.00000	0.00000	0.00030	0.03294

## Discussion

We here present a versatile, user-friendly graphical workflow system for modification calling to analyze RNA-Seq data. It can also be used to analyze any high-throughput data as long as they follow the formats listed in this technology report. Although this package allows creation and implementation of various workflows for processing and analysis, the application of this pipeline has limitations, which we would like to indicate hereafter and to point out possible solutions for adjustment.

### Limitations and Adjustability

The limitations of the workflow pertain mostly to the specific characteristics of the library preparation protocol. The workflow is tailored to the analysis of short RNA sequences, mostly tRNAs, and uses a “splice unaware” alignment because in the examples given, splicing is irrelevant. Accordingly, analysis of transcriptomic data should use an alignment tool that is specifically tailored to mapping of splice variants (“splice aware”).

Furthermore, algorithms such as the overhang trimming are not optimized for parallelization, which can lead to very long runtimes for the analysis, a problem potentially exacerbated by the large size of transcriptomic input data. Of course, as this Galaxy distribution makes use of the local computer’s processing power, large-scale analysis should not be performed on a device with weak computing capabilities. This Galaxy distribution, developed in a Unix environment, has not been tested on Windows platforms.

Detection efficiency of modified ribonucleotides is highly dependent on the dataset. tRNA samples show a high number of RT-impairing modifications, which can negatively affect the RT signals for surrounding positions, making it more difficult to detect modified positions of interest through filtering or machine learning. We also observed that detectability is highly dependent on read coverage. In some cases, modified low-coverage sites could not be detected as the RT signatures were noisy and thus not very pronounced. Moreover, the machine learning and prediction processes require an adequate number of training instances for a given modification. Modifications that are present only in low amounts are not compatible with the available machine learning process. Lastly, the workflow here presented was created and optimized to detect modifications, which naturally impair reverse transcription. However, this does not preclude modifications, which are made accessible for analysis through changes in the structural or chemical characteristics in a pretreatment by generating RT events like increased mismatch and arrest rates. Examples include the generation of RT signatures for *N* ^6^-methyladenosine (m^6^A) with an engineered polymerase with reverse transcriptase activity to induce mutations at m^6^A sites (Aschenbrenner et al., 2018), the enzymatic introduction of a bio-orthogonal propargyl group to trigger RT termination for m^6^A detection (Hartstock et al., 2018), and the site-specific installation of an allyl group to the *N* ^6^-position of adenosines, spontaneously inducing the formation of *N* ^1^,*N* ^6^-cyclized adenosine by iodination to create mutations to differentiate m^6^A, which is inert to allyl labeling, from adenosines at individual RNA sites (Shu et al., 2017).

While the available workflows were tailored toward our specific library preparation protocol and were created with the goal of detecting m^1^A, the workflows are easily adjustable for analysis of other modifications and other protocols. For example, the standard workflow also works without the overhang-trimming step, which allows the user to remove this step when using other library preparation protocols. In addition, the Galaxy interface allows for user-friendly customization of many input parameters. The customization is not limited to the software packages such as Cutadapt (Martin, 2011) and Bowtie (Langmead and Salzberg, 2012), but also includes individual Python scripts for the multiple workflows. Accordingly, adapter and barcode sequences can be replaced to fit the library preparation protocol, and other tasks like quality trimming can be performed. For the Python scripts, the range of adjustable parameters allows the user to change the modification of interest, filter criteria, features, and parameters for the machine learning model as well as several options for the visualization.

Furthermore, existing workflows can be easily rearranged to suit the desired analysis. The associated Galaxy toolshed allows for the installation of additional bioinformatics programs and enables the user to create entirely new workflows. For example, other alignment tools can be implemented that may improve or accelerate data processing or allow transcriptome-wide analysis for other data packages. In the provided tutorial, the installation of new software is described. As an example, we have incorporated the CUSHAW2 tool (Liu et al., 2012), which allows significant acceleration of the alignment speed, as a substitute for Bowtie 2. Our performance assessment showed that the alignment process could be sped up by a factor of up to six of the same datasets and on the same hardware platform. By reducing the time of the rather costly alignment step of the pipeline, it is possible to increase overall throughput. In return, the analysis of larger datasets is feasible within the same time in order to further increase the accuracy of the obtained results.

## Conclusion/Summary

Machine learning as an efficient tool for data mining is currently receiving enormous attention, which also extends to high-throughput sequencing data. Based on previous progress in machine learning for modification calling (Hauenschild et al., 2015), we here present a workflow that not only automatizes all steps, but which also, in principle, allows adaptation to “nonnatural” modifications, i.e. bioconjugate derivatives of RNA nucleotides after treatment with a chemical reagent or enzymes (Ofengand and Bakin, 1997; Carlile et al., 2014; Schwartz et al., 2014; Shu et al., 2017; Hartstock et al., 2018). In the course of development of reagent- and enzyme-based mapping procedures, repeated cycles of optimization, e.g. of reaction conditions, are necessary, but an assessment of modification calling performance for a given set of reaction conditions is extremely time consuming. The workflow here presents a solution to this bottleneck; while developed using the naturally occurring modification m1A as an example, it is conceived as such to be easily adaptable to the development of chemical reagents for modification mapping.

## Data Availability

The graphical workflow system, an instruction manual, and a tutorial are available at: https://github.com/HelmGroup, Repository: Galaxy_modification_calling.

Operating system(s): Linux, Programming language for custom scripts: Python, Other requirements: Docker (software) needs to be installed.

The AlkB test datasets analyzed and generated for this study can be found in the repository: Galaxy_modification_calling (https://github.com/HelmGroup/Galaxy_modification_calling/tree/master/TestData/AlkB).

Compressed files are provided in PKZIP and ZIP format and were compressed with 7-Zip.

Files: total_tRNA_yeast_untreated_R1.fastq (untreated yeast total tRNA – Read 1)

total_tRNA_yeast_untreated_R2.fastq (untreated yeast total tRNA – Read 2)

 total_tRNA_yeast_AlkB_treated_R1.fastq (AlkB-treated yeast total tRNA – Read 1)

total_tRNA_yeast_AlkB_treated_R2.fastq (AlkB-treated yeast total tRNA – Read 2)

 total_tRNA_yeast_untreated.profile (untreated yeast total tRNA – Profile)

total_tRNA_yeast_AlkB_treated.profile (AlkB-treated yeast total tRNA – Profile)

total_tRNA_yeast_reference.fasta (Reference total tRNA yeast)

Files for testing of the machine learning workflow can be found in the repository: Galaxy_modification_calling (https://github.com/HelmGroup/Galaxy_modification_calling/tree/master/TestData/Prediction).

Files: Known_m1A_sites_yeast (list of known m^1^A sites)

 total_tRNA_yeast_untreated.profile (untreated yeast total tRNA – Profile)

All other data are available from the corresponding authors upon reasonable request.

## Author Contributions

Conception and design: LS, SW, and MH; biomolecular experiments: SW, MK, and PJ; sequencing service: LA, VM, and YM; analysis and interpretation of the data: LS, SW, MK and MH; development and testing of the Galaxy modules: LS, TK, SN, BS, MK and AH; writing of the paper: LS, SW, and MH; proofreading and discussion: TS, BS, and AH.

## Funding

This work was supported by DFG grants HE3397/13-2, by DIP Grant RE 4193/1-1/RO 4681/6-1, and by JPND “RNA NEURO”/Bmbf grant FKZ: 01ED1804 and by the EPITRAN COST initiative (CA16120).

## Conflict of Interest Statement

The authors declare that the research was conducted in the absence of any commercial or financial relationships that could be construed as a potential conflict of interest.
